# Uniparental disomy - clinical consequences due to imprinting and activation of recessive genes

**DOI:** 10.1186/1755-8166-7-S1-I21

**Published:** 2014-01-21

**Authors:** Thomas Liehr

**Affiliations:** 1 Jena University Hospital, Friedrich Schiller University, Institute of Human Genetics, Kollegiengasse 10, D-07743 Jena, Germany

## 

Uniparental disomy (UPD) is often considered as an event to be characterized exclusively by molecular genetic or epigenetic approaches. Still in at least one third of cases UPD emerge in connection with or due to a chromosomal rearrangement.

Till date ~2,500 UPD cases detected in clinical, non-tumor cases are reported in the literature (http://www.fish.uniklinikum-jena.de/UPD.html). Based on this, the presently known imprinting syndromes, chromosomal contribution to UPD phenomenon, and cytogenetic subgroups of UPD and segmental UPD are reviewed.

UPD may arise in clinical cases, as well as it may be exclusively tumor related. For clinical cases imprinting is quantitatively the most important problem. Still isodisomy may also be a problem, due to “activation” of recessive mutation-events, thus inducing rare autosomal recessive disorders.

Overall, as UPD is more but an interesting rarity, the genetic background of each "UPD-patient" needs to be characterized besides by molecular methods, also by molecular cytogenetics in detail.

